# Co-Circulation of West Nile and Usutu Flaviviruses in Mosquitoes in Slovakia, 2018

**DOI:** 10.3390/v11070639

**Published:** 2019-07-12

**Authors:** Viktória Čabanová, Silvie Šikutová, Petra Straková, Oldřich Šebesta, Bronislava Vichová, Dana Zubríková, Martina Miterpáková, Jan Mendel, Zuzana Hurníková, Zdeněk Hubálek, Ivo Rudolf

**Affiliations:** 1 Slovak Academy of Sciences, Institute of Parasitology, Košice 040 01, Slovakia; 2 The Czech Academy of Sciences, Institute of Vertebrate Biology, Brno 603 65, Czech Republic

**Keywords:** *Culex* spp., mosquitoes, surveillance, Usutu virus, West Nile fever, West Nile virus

## Abstract

Monitoring West Nile virus (WNV) and Usutu virus (USUV) activity now has the highest priority among mosquito-borne pathogenic viruses circulating in the European Union. This study documents a first time detection and the co-circulation of WNV lineage-2 (with the minimal prevalence of 0.46%) and USUV clade Europe 2 (with the minimal prevalence of 0.25%) in mosquitoes from the same habitat of south-western Slovakia and underlines necessity to perform rigorous surveillance in birds, mosquitoes, horses and humans in that country.

## 1. Introduction

Recently, an increased number of arboviral outbreaks worldwide has led to the recognition of this group of pathogens as important health threats for humans and animals [1]. In Europe, there is growing concern about West Nile virus (WNV) infection. According to the European Centre of Disease Prevention and Control (ECDC), the number of reported human autochthonous infections due to WNV lineage 2 (WNV-2) increased 7.2-fold in 2018 in comparison to the previous season. A total of 180 fatal human cases were reported in Greece, Italy, Romania, Serbia, Kosovo, Turkey, Bulgaria, the Czech Republic and Hungary [2]. Mosquitoes from the genus *Culex* acquire WNV when feeding on birds. Human and horses are considered dead-end hosts. In humans, WNV infection is mainly asymptomatic. However, 20% to 40% of cases may lead to influenza-like symptoms, and 1% could develop to serious neuroinvasive disease [3].

Widespread activity by Usutu virus (USUV) has also been observed, with *Culex* spp. serving as its main vector in the field. Since its introduction to Europe in 1996, USUV has rapidly expanded, causing epizootics among wild and captive birds in central (Czech Republic, Austria, Hungary), south (Italy) and western (Netherlands, Germany, Belgium) Europe, with mass mortalities reported, particularly in the blackbird (*Turdus merula*) population. The first human cases of severe USUV encephalitis, documented in immunocompromised patients from Italy in 2009, confirmed the zoonotic potential of this virus. Importantly, USUV has been occasionally detected in asymptomatic blood donors throughout Europe, and several USUV infections in immunocompetent patients have been reported by recent studies [4]. According to phylogenetic analyses of NS5 genomic region of the European USUV strains, up to seven lineages (Europe 1–5, Africa 2–3) now circulate in Europe [5].

Historically, the circulation of WNV was reported several times in different geographical areas of Slovakia. Antibodies against the virus were detected in human samples from Michalovce (4%) and Nitra regions (4%), where 8.5% of WNV seroprevalence was also confirmed in cattle [6,7]. WNV monitoring then continued along the rivers Ipeľ, Rimava and Bodva at Slovak-Hungarian borders of Central and Eastern Slovakia. Three strains of WNV were isolated from the migrating birds, such as the Green Sandpiper (*Tringa ochropus*), Lapwing (*Vanellus vanellus*) and Turtle Dove (*Streptopelia turtur*) and were identified by a haemagglutination inhibition assay [8]. The last comprehensive study of the twentieth century was performed in sheep farms of Eastern Slovakia, and WNV antibodies were detected in 1% of sheep [9]. More recently, serosurveys by Hubálek et al. [10] and Csank et al. [11,12,13] provided new insights into WNV circulation in horses and birds within Slovakia. According to these studies, a prevalence of WNV antibodies ranged between 11.7% to 11.9% in birds [12,13] and 8.3% to 11.7% in horses [10,12]. The majority of infected horses came from the south-western Slovakia region. Furthermore, a RNA of WNV-2 was confirmed in 21.8% and 26.7% of tested birds from different parts of Slovakia [11].

The presence of USUV in Slovakia was recently indicated by the discovery of USUV antibodies in one Great Tit (*Parus major*) from Levice district (Nitra region) and in 16 green lizards (*Lacerta viridis*) captured in Slovak Karst National Park [12,14].

The confirmation of WNV circulation in field-collected mosquito vectors has been reported only once from Slovakia, in 1972, when WNV was isolated from a pool of field-collected *Aedes cantans* from Malacky (western Slovakia) and identified using a combination of haemagglutination inhibition, complement fixation and virus neutralisation tests [15]. Importantly, WNV-2 and USUV activity is documented in neighbouring Hungary, Austria and the Czech Republic. Thus we aimed at molecular screening of local mosquitoes for WNV-2 and USUV to consider public health risk in the region.

## 2. Materials and Methods

### Mosquito Sampling and Molecular Screening

**Figure 1:**
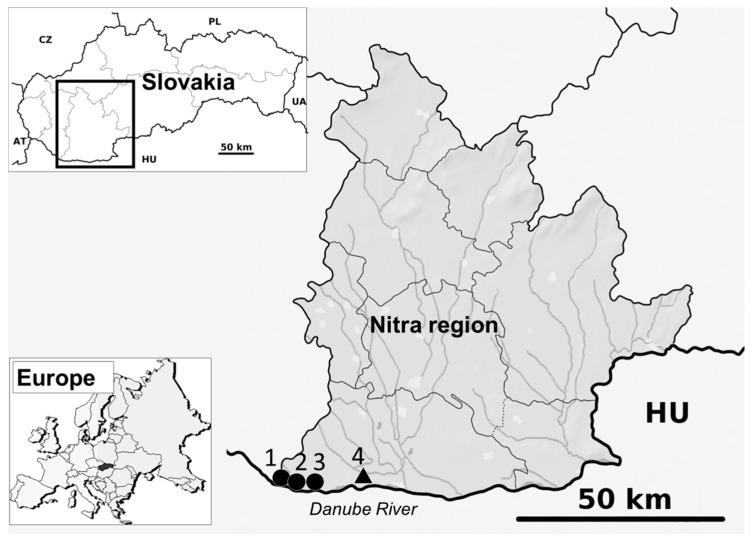
Locations of four study sites for mosquito trapping, Nitra region, Slovakia. A long-term study locality is marked by a triangle, the short-term study localities are marked by circles (1 = Čičov, 2 = Kližská Nemá, 3 = Veľké Kosihy, 4 = Komárno-Nová Stráž).

The Komárno district (Nitra region) in the south-western part of the country (Figure 1) was selected for the survey. The Nitra region was previously identified as an area with WNV occurrence, based on serosurveys [6,7,10]. Mosquito collection was provided by two different sampling protocols: the long-term part of the study was performed in the suburb of Komárno city–Nová Stráž between June and November 2018. The BG-Mosquitaire CO_2_ Mosquito trap (Biogents, Regensburg, Germany) was set up on private property containing a small farm with sheep and poultry. For the second part of the screening, wetland and fishpond areas (*n* = 3) in the Komárno district were selected. Mosquitoes were caught by EVS CO_2_ Mosquito traps (BioQuip Products, Inc., Rancho Dominguez, CA, USA) accompanied with dry ice during two field samplings—first at the end of July, and second in the middle of September 2018. Immediately after collection, trapped mosquitoes were transported to the laboratory in cooled containers, killed at −20 °C and stored at −80 °C until further processing. All other steps were performed on ice. Females were identified by morphological features [16] and pooled in groups of a maximum of 25 (long-term study) or 50 (short-term study) individuals per tube. Pools were homogenised with a 200 to 800 µl of sterile PBS and a 5 mm stainless beads in Qiagen Tissue Lyser (Qiagen, Hilden, Germany). RNA extraction was performed as previously described by Rudolf et al. [17]. Flaviviral RNA was detected by a conventional reverse transcription-polymerase chain reaction (RT-PCR), according to Scaramozzino et al. [18]. Flavivirus-positive PCR samples were further amplified by specific RT-PCR for WNV and USUV [18,19] and sequenced by the Sanger method. The sequences were compared by a Basic alignment search tool (BLAST) and further aligned with partial nucleotide sequences of the particular protein coding region and/or complete genome sequences deposited in the GenBank database. Phylogenetic analyses were conducted using the Maximum Likelihood (ML) algorithm using the Kimura-2 model (MEGA 6.0). The stability of the tree topologies was tested by bootstrap resampling of 1000 replicates. Furthermore, in a case of a PCR sample with a strong positive signal, the homogenates were inoculated intracerebrally (i.c., 20 μL) in specific pathogen free (SPF) suckling ICR (outbred) mice with the aim to isolate the virus [19]. The experiment with laboratory mice was conducted in accordance with the Czech Animal Protection Act no. 246/1992.

## 3. Results and Discussion

A total of 2817 mosquitoes from eight different taxa (*Anopheles hyrcanus*, *Anopheles maculipennis* s.l., *Anopheles plumbeus*, *Coquillettidia richiardii*, *Culex modestus*, *Culex pipiens* complex, *Culiseta annulata* and *Uranotaenia unguiculata*) were divided into 226 pools and examined for flaviviral RNA (Table 1 and Table 2).

**Figure 2:**
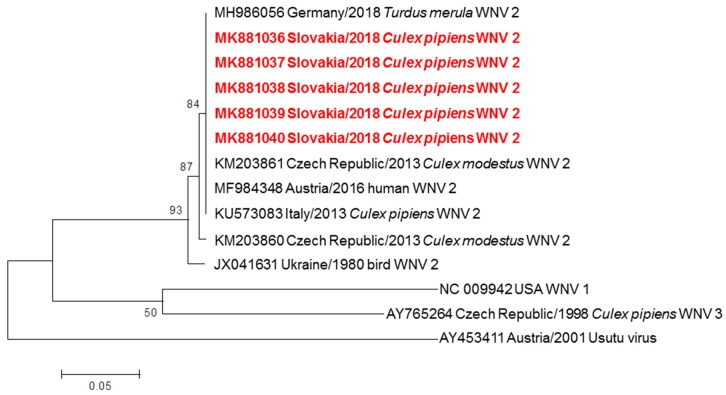
Phylogram demonstrating the relationship of WNV extracted from mosquitoes in Slovakia, based on 305 bp long partial nucleotide sequence of the envelope gene (position 1531–1836) from Slovak sequences obtained in this work and other WNV strains circulating in Central Europe. Each record consists of particular accession number, place and year of detection/isolation and source (human/mosquito/bird). Slovak WNV sequences are highlighted in red. Phylogenetic analyses were conducted using Maximum Likelihood (ML) algorithm using the Kimura-2 model (MEGA 6.0). The robustness of trees was tested by bootstrap resampling of 1000 replicates, and its values are listed near the nodes. The horizontal bar shows genetic distance.

WNV specific RNA was detected in 13 pools, minimum prevalence rate reached 0.46% in all examined mosquitoes (2 positive pools originated from *Cx. modestus*, 10 positive pools originated from *Cx. pipiens* complex and one pool of females originated from unidentified *Culex* spp. damaged during sampling). A BLAST analysis of sequences showed 99.48% to 100% identity with the Central European lineages WNV-2 from Germany (MH986056), Italy (KU573083) and Austria (MF984348). A phylogram confirmed the genetic relationship of Slovak WNV strains with lineage 2 (Figure 2). The closest relationship was recognised with German (MH986056), Austrian (MF984348), Italian (KU573083) strains and one Czech strain (KM203861). The nucleotide sequences of these WNV strains from Slovakia are available in the GenBank database with accession numbers MK881036 to MK881040. One pool #SK-1 (50 females of *Cx. pipiens* collected at Velké Kosihy fishpond on September 12, 2018), tested positive for WNV-2 by RT-PCR, was inoculated intracerebrally to suckling mice. The bacteriologically sterile mosquito suspension killed 9 of 10 inoculated suckling mice after 5 days; average survival time (AST) of SM was 5.0 days, and the brain suspension of infected mice contained WNV-2.

**Figure 3:**
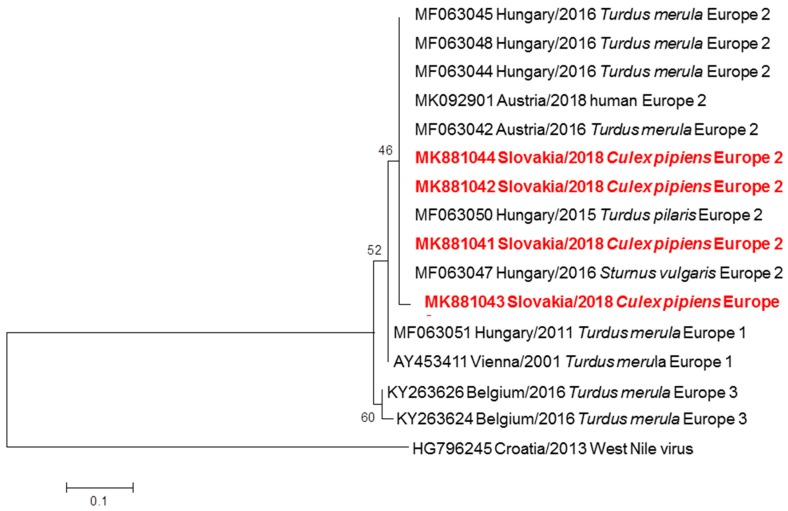
Phylogram demonstrating the relationship of USUV extracted from mosquitoes in Slovakia, based on 512 bp long partial nucleotide sequence of the NS5 gene (position 9177–9689) from Slovak sequences obtained in this work and other USUV strains circulating in Central Europe. Each record consists of particular accession number, place and year of detection/isolation and source (human/mosquito/bird). Slovak USUV sequences are highlighted in red. Phylogenetic analyses were conducted using Maximum Likelihood (ML) algorithm using the Kimura-2 model (MEGA 6.0). The robustness of trees was tested by bootstrap resampling of 1000 replicates, and its values are listed near the nodes. The horizontal bar shows genetic distance.

Seven pools of *Cx. pipiens* complex yielded positive USUV sequences, with the minimal prevalence of 0.25% in all examined mosquitoes. A BLAST analysis revealed 99.80% to 100% identity of Slovak USUV strains with isolates recovered from blackbirds, one starling and one fieldfare in Hungary (MF063044-48; MF063050) and Austria (MF063042). The ML algorithm placed the Slovak strains into a common cluster with the Hungarian (MF063044-46; MF063048) and Austrian (MK092901; MF063042) strains and showed relatedness to USUV Europe 2 according to Aberle et al. [20] (Figure 3). The isolates of Slovak USUV strains from mosquitoes were submitted to the GenBank database with accession numbers MK881041 to MK1044.

All positive mosquitoes for WNV and USUV were trapped between July 10 and September 14, 2018. Co-circulation of USUV and WNV was observed in the suburban habitat of Komárno, Nová Stráž, on private property with a small farm. *Cx. pipiens* serves as a vector for both viruses, and their co-infection were observed in five pools.

Forty-five years after the last report [15], the presence of WNV in mosquitoes is documented in Slovakia again. In addition, USUV RNA was detected in mosquitoes from this country for the very first time. In accordance with previous data [6,7,8,9,10,11,12,13,14,15], autochthonous occurrence of both flaviviruses was confirmed in this study. Slovakia, especially the Nitra region, should be considered a high risk area for WNV and USUV circulation in Europe. Interestingly, no human cases have been reported so far from Slovakia, which might be due to underreporting of the disease in the country or the misdiagnosis of WNV and USUV cases as central nervous system infections of unknown origin. The mosquitoes *Cx. pipiens* and *Cx. modestus* serve as vectors of WNV and *Cx. pipiens* as a vector of USUV in Komárno district and these viruses were observed to co-occur in the same habitat, similar to previous findings in South Moravia and other European countries [19,21]. This extended geographic range of co-circulation of the two viruses is probably due to the movements of the wild bird reservoir hosts. Furthermore, the lineages WNV-2 and USUV Europe 2 discovered during this study are closely related to strains detected in humans in Austria [20]. This means the Slovak strains are likely also capable of causing human infections. It is worth mentioning the remarkably high prevalence of WNV in Slovak mosquitoes caught in this study in comparison to the neighbouring Czech Republic or Hungary, which may indicate potentially increased number of human autochthonous cases in the coming WNV season. This study brings very important data which should alert general practitioners, infectious disease specialists, veterinary doctors and epidemiologists about the presence of active WNV and USUV circulation in mosquitoes in Slovakia. It is suggested that public health authorities should implement WNV surveillance activities (in mosquitoes, birds, horses and humans) in other parts of the country as well.

## Figures and Tables

**Table 1:** Mosquito sampling by BG-Mosquitaire in Komárno suburb—Nová Stráž between June and November 2018 (pool—max. 25 individuals).

Species	Individuals/Pools Tested	WNV+	USUV+
*Anopheles hyrcanus*	1/1	ND	ND
*Anopheles maculipennis* s.l.	63/19	ND	ND
*Anopheles plumbeus*	2/2	ND	ND
*Anopheles* spp.	3/3	ND	ND
*Coquillettidia richiardii*	397/29	ND	ND
*Culex modestus*	69/20	ND	ND
*Culex pipiens* complex	1036/68	7	6
*Culex* spp. *	178/14	1	1
*Culiseta annulata*	10/7	ND	ND
**Total**	**1759/163**	**8**	**7**

Legend: s.l.—sensu lato; spp.—species in that given genus; ND—virus not detected; * damaged individuals identified only to genus level; WNV—West-Nile virus; USUV—Usutu virus.

**Table 2:** Mosquito sampling by EVS traps situated in wetland and fishpond area of Komárno district, July and September 2018 (pool—max. 50 individuals).

**Study Site: Čičov**			
**Species**	**Individuals/Pools Tested**	**WNV+**	**USUV+**
*Anopheles hyrcanus*	8/1	ND	ND
*Anopheles plumbeus*	1/1	ND	ND
*Culex modestus*	26/1	ND	ND
*Culex pipiens* complex	135/3	1	ND
*Uronatenia unguiculata*	1/1	ND	ND
**Subtotal**	**171/7**	**1**	**0**
**Study Site: Kližská Nemá**			
**Species**	**Individuals/Pools Tested**	**WNV+**	**USUV+**
*Anopheles hyrcanus*	4/1	ND	ND
*Anopheles maculipennis* s.l.	3/1	ND	ND
*Culex modestus*	150/3	ND	ND
*Culex pipiens* complex	80/2	1	ND
**Subtotal**	**237/7**	**1**	**0**
**Study Site: Veľké Kosihy**			
**Species**	**Individuals/Pools Tested**	**WNV+**	**USUV+**
*Anopheles hyrcanus*	112/8	ND	ND
*Anopheles maculipennis* s.l.	54/7	ND	ND
*Anopheles* spp.	1/1	ND	ND
*Coquillettidia richiardii*	12/6	ND	ND
*Culex modestus*	204/9	2	ND
*Culex pipiens* complex	252/11	1	ND
*Culex* spp. *	6/2	ND	ND
*Culiseta annulata*	6/4	ND	ND
*Uranotaenia unguiculata*	3/1	ND	ND
**Subtotal**	**650/49**	**3**	ND
**Total**	**1058/63**	**5**	**0**

Legend: s.l.—sensu lato; spp.—species in that given genus; ND—virus not detected; * damaged individuals identified only to genus level; WNV—West-Nile virus; USUV—Usutu virus.
